# Clinical stabilization of a highly refractory acute myeloid leukaemia under individualized treatment with immune response modifying drugs by in vivo generation of dendritic cells of leukaemic origin (DCleu) and modulation of effector cells and immune escape mechanisms

**DOI:** 10.1186/s40364-025-00817-8

**Published:** 2025-08-15

**Authors:** Giuliano Filippini Velázquez, Philipp Anand, Joudi Abdulmajid, Xiaojia Feng, Jan Frederic Weller, Klaus Hirschbühl, Helga Schmetzer, Christoph Schmid

**Affiliations:** 1 Section for Stem Cell Transplantation and Cellular Therapy Research, Department of Hematology and Oncology, Augsburg University Hospital and Medical Faculty, Stenglinstr. 2, D-86156 Augsburg, Germany; 2 Department of Medicine III, University Hospital of Munich, 81377 Munich, Germany; 3 Department of Hematology and Oncology, University Hospital Hamburg Eppendorf, Hamburg, Germany; 4 Bavarian Cancer Research Center (BZKF), Comprehensive Cancer Center, Augsburg, Germany

## Abstract

The conversion of leukemic blasts into antigen-presenting dendritic cells of leukemic origin (DC_leu_) by GM-CSF and PGE1 has demonstrated preclinical efficacy in eliciting leukaemia-specific immune responses, offering a promising immunotherapeutic strategy for relapsed/refractory AML. We report on a 65-year-old patient with AML refractory to multiple treatment lines, including two allogeneic stem cell transplantations, who received individualized experimental treatment with intravenous GM-CSF and PGE1 and no additional anti-leukaemic therapy. Based on preceding ex-vivo treatment of patient´s blood with GM-CSF/PGE1 that showed immune activation and blast lysis, we hypothesized that intravenous administration of the compounds to the patient would promote in-vivo antileukaemic immune reactions and potentially induce clinical response. Eight treatment cycles were administered, and extensive immune monitoring was performed. The treatment was well tolerated and resulted in sustained clinical stabilization over four months. Immune monitoring showed generation of mature DC_leu_, activation of leukaemia-directed effector and memory cells (including IFN-γ–producing and degranulating T and NK cells), downregulation of immune checkpoint (PD-1/CTLA-4) expressing T cells and blasts, and a reduction in regulatory B- and T cells. This case illustrates the feasibility and tolerability of GM-CSF + PGE1 therapy and its potential to modulate anti-leukaemic immunity in a patient with highly refractory AML.

**Supplementary Information:**

The online version contains supplementary material available at 10.1186/s40364-025-00817-8.

**To the editor**

Allogeneic stem cell transplantation (alloSCT) remains the most effective curative approach for high-risk acute myeloid leukaemia (AML). Nevertheless, 30–60% of patients relapse post-transplant, frequently based on defined immune escape mechanisms, including mismatched HLA loss, regulatory T-/B-cell expansion, or upregulation of immune checkpoints (PD-1/CTLA-4/TIM-3) on effector cells [1–3].

Dendritic cell (DC)-based immunotherapy may circumvent immune escape [4]. We previously demonstrated that leukaemia derived DCs (DC_leu_) can be generated ex-vivo using the immune response modifiers GM-CSF and PGE1 (termed “Kit-M”) and induce leukaemia-specific B-, T-, and NK cell responses in preclinical models [5, 6]. Here we report the clinical translation of this approach in a patient with refractory AML.

A 65-year-old male with secondary AML (adverse genetics: *BCR::ABL1*,* ASXL1*,* KRAS*,* RUNX1*,* FLT3-ITD*,* IKZF1*), initially treated with standard induction and alloSCT in MRD-positive first complete remission, presented with haematological and extramedullary (malignant pleural effusions requiring paracentesis 2-3x/week) relapse after multiple lines of therapies, including a second haplo-identical alloSCT six months earlier. Donor lymphocyte infusions were precluded due to genomic HLA loss in leukaemic blasts [1], and the leukaemia was refractory to Azacitidine salvage therapy. A detailed description of the treatment history is provided in Supplemental Fig. 1.

In the absence of approved treatment options, an individualized immunotherapeutic approach using Kit-M was discussed, thereby re-purposing two compounds approved for clinical use in other indications [7]. Previously, successful generation of DC_leu_ subsets had been demonstrated after stimulation of the patient´s blood with Kit-M. Beyond, mixed lymphocyte cultures had confirmed activation of effector and memory T- and NK cells, downregulation of regulatory T cells (Treg), and induction of blast-directed cytotoxicity. Based on this observation, we hypothesized that systemic administration of the compounds of Kit-M to the patient would promote in-vivo antileukemic immune reactions and potentially induce clinical response. The patient was extensively informed about the experimental treatment nature. Following written consent for both treatment and sequential collection of blood samples for immune monitoring; and following approval by the patient’s health care provider and the local ethics committee (LMU #33905), Kit-M treatment was administered in eight 5-day cycles over a total 105 days, with dose escalation during the initial four cycles to ensure safety (Table 1).

Treatment was well tolerated; no serious adverse events or graft-versus-host disease were observed. The patient´s clinical status remained stable (Karnofsky performance score 90). Red cell transfusion requirements decreased by 25% compared to the prior treatment phase, and frequency of pleural paracentesis could be decreased to ≤ 1x/week. Peripheral leucocyte (WBC) and blast counts remained stable (median: WBC: 2.28/nl; blasts: 9%), indicating controlled leukaemia burden. Hence, Kit-M seemed to support granulopoiesis without promoting blast proliferation. After four months, AML progressed (peripheral blood [PB] blasts: >40%), prompting Kit-M discontinuation. Palliative chemotherapy was initiated, and the patient died four weeks later from disease progression (Supplemental Figs. 2–4 illustrate the clinical course and peripheral cell counts during treatment).

Comprehensive immune monitoring during treatment revealed sustained increases in mature DCs and DC_leu_ in PB (Supplemental Fig. 5). Concurrently, activation of both innate and adaptive immune compartments was observed, including expansion of IFN-γ + memory γδ T cells, de-granulating cytotoxic T-/NK cells, and invariant NKT cells. TH1 polarization increased, while TH2 and Treg frequencies declined. Notably, frequencies of effector T-cells expressing inhibitory checkpoint receptors PD-1, CTLA-4, and KLRG-1 were markedly decreased (Fig. 1). Regulatory B cells were downregulated, while memory B cells expanded (Supplemental Fig. 6).These changes imply a systemic immunologic reprogramming toward a more activated state. Supplemental Table **1** shows cell subtypes analysed in this study.

Importantly, while stable during Kit-M administration, PB blast counts increased during treatment breaks, suggesting that continuous immune activation contributed to temporary leukaemic containment. Despite pre-existing HLA loss on malignant blasts, leukaemia-directed immune responses were observed, potentially either due to targeting of HLA-retaining subclones or involved (HLA-independent) NK-mediated mechanisms. Beyond generation of DC_leu_, extensive immune monitoring demonstrated a broader immune stimulation by Kit-M, involving NK cell stimulation, reversal of T cell exhaustion and inhibition via immune checkpoints, whereby the relative role of the different mechanisms to clinical effects remains to be defined. Nonetheless, final disease progression occurred. Decrease of mature DC (corresponding to inefficient antileukaemic functionality) and increase of immune checkpoints on T cells and blasts (2B4, TIM-3, data not shown) suggested the emergence of various immune escape mechanisms.

This case illustrates the potential of Kit-M to elicit leukaemia-reactive immune responses, even in heavily pre-treated patients, that were able to stabilize both haematologic and extramedullary disease. Whereas in general the approach might represent a novel strategy to be integrated into the treatment of AML, effects of Kit-M monotherapy were transient, and insufficient to induce durable disease control in this highly proliferative disease. Unlike traditional DC-based strategies such as vaccines, requiring intensive ex-vivo manipulation, Kit-M induces DC_leu_ in-vivo, thereby avoiding the need of complex cell manufacturing [8]. Its favourable safety profile and profound immunologic effects support further investigation [5]. We hypothesize that the anti-leukaemia efficacy may be enhanced through rational combination with other immunomodulatory agents (e.g., hypomethylating agents or checkpoint inhibitors), earlier application (MRD-positive remission), or continuous subcutaneous delivery to avoid treatment interruptions.

**Table 1:** Experimental treatment protocol of Kkit M (GM-CSF + PGE1). PGE1: Prostaglandin E1, GM-CSF: Granulocyte-Macrophage Colony-Stimulating Factor. i.v.: intravenous

Phase	Day	Drug	Dosage (i.v.)	Schedule
**Ramp up phase**	0–2	GM-CSF PGE1	75 µg/m² 20 µg	4 h (9 am − 1 pm) 2 h (1 pm − 3 pm)
	3–4	GM-CSF PGE1	75 µg/m² 40 µg	4 h (9 am − 1 pm) 2 h (1 pm − 3 pm)
	7–11	GM-CSF PGE1	75 µg/m² 40 µg	4 h (9 am − 1 pm) 2 h (1 pm − 3 pm)
	15–19	GM-CSF PGE1	75 µg/m² 40 µg	4 h (9 am − 1 pm) 2 h (1 pm − 3 pm)
	22–26	PGE1 GM-CSF PGE1	40 µg 75 µg/m² 40 µg	2 h (9 am − 11 am) 4 h (11 am − 3 pm) 2 h (3 pm − 5 pm)
**Treatment break** ^*^				
Final dose**	51–55	PGE1 GM-CSF PGE1	40 µg 75 µg/m² 40 µg	2 h (9 am − 11 am) 4 h (11 am − 3 pm) 2 h (3 pm − 5 pm)

* prolonged by one week due to minor surgery

** repeated in five-day cycles from d71-75, d87-91, and d101-105, slight differences in the intervals due to patient’s preference

**Fig. 1:**
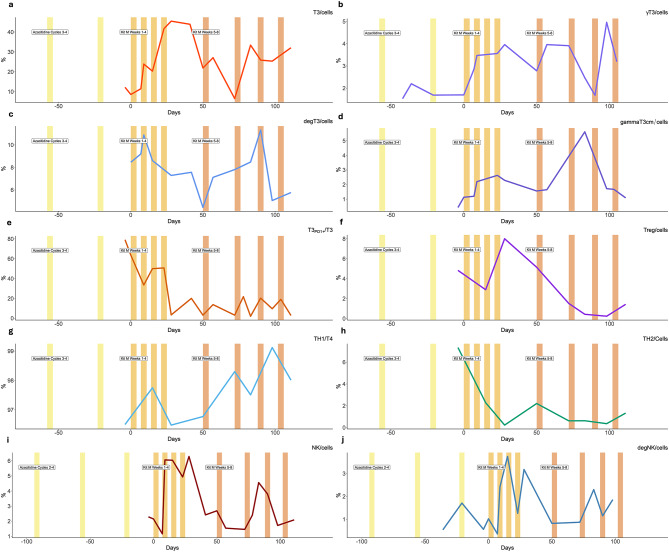
**a-j**. Relative frequencies of T cells, NK cells and subpopulations in peripheral blood during treatment. The bars in colors represent treatment phases: every yellow bar indicate 1 cycle of Azacitidine; orange bars (starting on day 0) represent the ramp up phase of experimental treatment with Kit M (each bar indicate one five-day cycle with daily infusion of *Kit M*: GM-CSF + PEG1); brown bars (starting day 50) indicate the final dose phase of Kit M (each bar d1-5 with daily infusions). For more details on the treatment protocol see Table 1. (**a**) T cells (T3), (**b**) Interferon gamma (IFN)-producing T cells (gammaT3), (**c**) Degranulating (CD107a+) T cells (degT3), (**d**) IFNy-producing central memory T cells (gammaT3cm), (**e**) T cells expressing PD-1 (T3_PD1_), (**f**) Regulatory T cells (Treg), (**g**) T helper 1 cells (TH1), and (**h**) T helper 2 cells (TH2), (**i**) Natural killer cells (NK), (**j**) degranulating (CD107a+) NK cells (degNK). The colored lines illustrate the course of relative frequencies (%) of the given cell subtype measured at different time points during treatment course

## Supplementary Information

Below is the link to the electronic supplementary material.

Supplementary Material 1

## Data Availability

All data generated or analysed during this study are included in this published article (and its supplementary information files).
