# Supplementary material for: Clinical stabilization of a highly refractory acute myeloid leukaemia under individualized treatment with immune response modifying drugs by in vivo generation of dendritic cells of leukaemic origin (DCleu) and modulation of effector cells and immune escape mechanisms
Source: Biomark Res. 2025 Aug 15;13:104. doi: 10.1186/s40364-025-00817-8 (PMC12357330; doi:10.1186/s40364-025-00817-8)
Supplement: Supplementary file 1 — Supplementary Material 1 [file 40364_2025_817_MOESM1_ESM.docx]

**Supplement to the letter** by G. Filippini Velázquez, P. Anand et al.: *“Clinical Stabilization of a Highly Refractory Acute Myeloid Leukaemia Under Individualized Treatment with Immune Response Modifying Drugs by In Vivo Generation of Dendritic Cells of Leukaemic Origin (DCleu) and Modulation of Effector Cells and Immune Escape Mechanisms”*

**Supplemental methods**

To ensure safety, strict criteria had been established for discontinuing the experimental treatment: 1) Immediate termination of infusion for both drugs in the presence of any toxicities > CTC grade 2, with the resumption of infusions contingent on the complete normalization of relevant parameters. 2) Termination of GM-CSF therapy if the peripheral blast counts exceeded 10000/µl or surpassed 50% of leukocytes, or in the event of lung-toxicities (severe dyspnoea, significant alterations in blood gas parameters). 3) Discontinuation of PGE-1 therapy in case of a decrease in blood pressure (<100mmHg systolic or 50mmHg diastolic) or manifestation of clinical signs of cardiac failure (dyspnoea, pulmonary or peripheral oedema).

**Functional tests and Immune monitoring**

Peripheral blood (PB) and bone marrow (BM) samples for specific immune monitoring were collected in lithium heparin tubes by the investigators at the University Hospital Augsburg at regular intervals over 9 months.

*Ex-vivo analyses of patient´s whole blood samples:* before experimental treatment initiation, peripheral mononuclear (PBMC)/T cells were isolated and frozen for mixed lymphocyte culture (MLC); Dendritic cell cultures (DCC) were performed from the patient’s heparinized whole blood (WB). *Ex- vivo* generation of DC/DCleu from the patient’s blood was monitored and quantified as previously described. To investigate the ability of effector cells after MLC to lyse target cells, the cytotoxicity fluorolysis (CTX) assay was conducted. *(Schwepke, et al., International Journal of Molecular Sciences 2022, Klauer et al., Transfusion Medicine and Hemotherapy 2021, Amberger et al., International Journal of Molecular Sciences, 2019).*

*Immune Monitoring*: Quantification of various cell populations before, during, and after treatment as well as of cells after culture was conducted through flow cytometry. *(Amberger et al., International Journal of Molecular Sciences, 2019)* To evaluate and quantify IFNγ producing cells and CD107a degranulating cells in PB from the patient during treatment as well as after MLC, intracellular interferon (INZ) and degranulation (DEG) assays were performed. *(Schutti et al., International Journal of Molecular Sciences* 2024) For more details see **Supplemental table 1.**

Sample analyses were performed in accordance with the declaration of Helsinki, after approval by the local ethics committee (Ludwig-Maximilians-Universität; no. 33905).

**Dendritic cell culture (DCC) and mixed lymphocyte culture (MLC)**

DC cultures from patient´s WB were harvested on day 7-8 and MLC cultures were set up with 1x${10}^{6}$(previously frozen) T cells from the AML-patient and co-cultured with approximately 2.5x${10}^{5}$ cells from DC/DCleu generating cultures (DCWB(Control), DCWB (Kit M)) and RPMI in a total of 1 ml in the presence of IL-2 as shown before *(Amberger et al., International Journal of Molecular Sciences, 2019).* Different immune cell subtypes after MLC were quantified by flow cytometry *(Klauer et al., Transfusion Medicine and Hemotherapy, 2021).*

**Detection of antigen specific cells using an intracellular Interferon γ (INZ) and a degranulation assay (DEG)**

Primary goal of the degranulation assay (DEG) was to evaluate lysosomally-associated membrane protein 1 (LAMP-1, CD107a) expression in various lymphocyte subsets, serving as a marker for assessing degranulation activity and cytotoxicity induction. IFN-γ is primarily produced by NK cells and T cells, playing a crucial role in activating antitumor immune responses. Using the INZ assay, we quantified the extent of IFN-γ-producing cells. The assays were conducted on uncultured PB cells as well as after MLC. Additional stimulation of potentially leukemia specific cells was achieved using leukemia-associated antigens (LAAs) such as Wilms Tumor-1 (PepTivator WT1) and Preferentially Expressed Antigen of Melanoma (PepTivator PRAME) as shown before *(Schutti et al., International Journal of Molecular Sciences* 2024).

**Cytotoxicity fluorolysis (CTX) assay**

To investigate the ability of effector cells to lyse target cells, the cytotoxicity fluorolysis (CTX) assay was conducted. In this assay, T cell enriched cells are stimulated with GM-CSF+PEG1-treated (vs not pretreated) WB after MLC to lyse thawed viable patient´s blast containing PBMC (target cells) stained with two different blast markers. The lytic activity of effector cells was calculated as the frequencies of viable target cells in the culture with co-cultured effector and target cells (for 3h and 24h) compared to control.

**Characterisation of the cells by flow cytometric analysis**

Panels were designed using monoclonal antibodies from Beckman Coulter (Krefeld, Germany), Becton Dickinson (Heidelberg, Germany), BioLegend (Amsterdam, Netherlands), Miltenyi Biotec (Bergisch Gladbach, Germany), and Santa Cruz Biotechnology (Heidelberg, Germany) conjugated with FITC (fluorescein isothiocyanate), PE (phycoerythrin), PE/Cy 7 (phycoerythrin/cyanine 7), or APC (allophycocyanin). For intracellular staining, FIX&PERMT Cell Fixation and Permeabilisation Kit (ThermoFisher Scientific, Darmstadt, Germany) was used. The flow cytometric analyses were conducted using a FACSCaliburTM flow cytometer, and data analyses were performed utilizing the Cell-Quest data acquisition and analysis, software from Becton Dickinson, Heidelberg, Germany.

**Clinical history (see also Supplemental figure 1)**

We report the case of a 65-year-old man with the diagnosis of a secondary acute myeloid leukaemia (sAML) that relapsed 6 months after a second allogeneic hematopoietic stem cell transplantation (alloSCT).

The patient presented initially (06/2020) with a *BCR/ABL1* positive chronic myeloid leukaemia. Nilotinib, a tyrosine-kinase inhibitor (TKI) in standard dosage was started. Four months later (10/2020) a blast crisis was suspected, but a bone marrow (BM) biopsy revealed a sAML, based on the acquisition of new molecular markers besides *BCR/ABL1* (*ASXL1*, *KRAS*, *RUNX1*, and *FLT3-ITD*). The patient was enrolled in the “DaunoDouble” clinical trial and received two cycles of standard “7+3” induction treatment with Cytarabine/Daunorubicin. In addition, Midostaurin was given on days (d) 8-21 for *FLT3-ITD.* Complete remission (CR) without detection of myeloid markers and only minimal residual disease (MRD) persistence by detection of *BCR/ABL-*1 (ratio: 0.002) was achieved.

He received the first alloSCT from a haploidentical sibling (03/2021) after myeloablative conditioning and post-transplant Cyclophosphamide (pTCY). Sorafenib maintenance was started on d128 but discontinued after graft-versus-host disease (GvHD). He remained in molecular CR for 8 months until *BCR/ABL1* was newly detected in peripheral blood (PB). A BM-biopsy showed morphological CR, but MRD was confirmed (*BCR/ABL1* only)*.* Pre-emptive treatment with Dasatinib was given until 1 month later, leukemic blasts were detected in BM with increase in *BCR/ABL1* levels and reappearance of *RUNX1.* Dasatinib was stopped and treatment for AML relapse with Azacitidine/Venetoclax was started. After 2 cycles of refractory treatment, a second alloSCT from his haploidentical son was performed (06/2022). We started prophylactic treatment with Dasatinib because of the last detection of BCR/ABL and disappearance of the FLT3-ITD clone. After this, he remained in remission for 6 months. On 12/2022 AML relapsed with detection of classical myeloid molecular markers (*KRAS*, *RUNX1*, *FLT3-ITD*, *DNMT3A*) without detection of *BCR/ABL-1,* and confirmation of HLA loss.

Treatment for relapse with dose reduced Azacitidine was initiated. Venetoclax was not given due to persistent cytopenia. By this time, repeated paracentesis for recurrent pleural effusions was required. Initially, immunophenotyping and molecular analyses failed to detect AML involvement, and TKI-related serositis was suspected. Finally, after serial analyses extramedullary leukaemia was confirmed.

The leukaemia persisted despite four cycles Azacitidine. NGS-analyses of BM revealed persistence of *RUNX1* and gain of *IKZF1* mutation, but no *FLT3-ITD* or *BCR/ABL1*. On 04/2023 experimental treatment with Kit-M (GM-GCSF 75µg/m2 + PGE 80µg d1-5) was started. This treatment was administered over a period of 11 weeks. On 08/2023, the AML progressed again, and after 1 cycle of Decitabin/Venetoclax the patient died of leukaemia-progression.

**Supplemental figure 1**

**
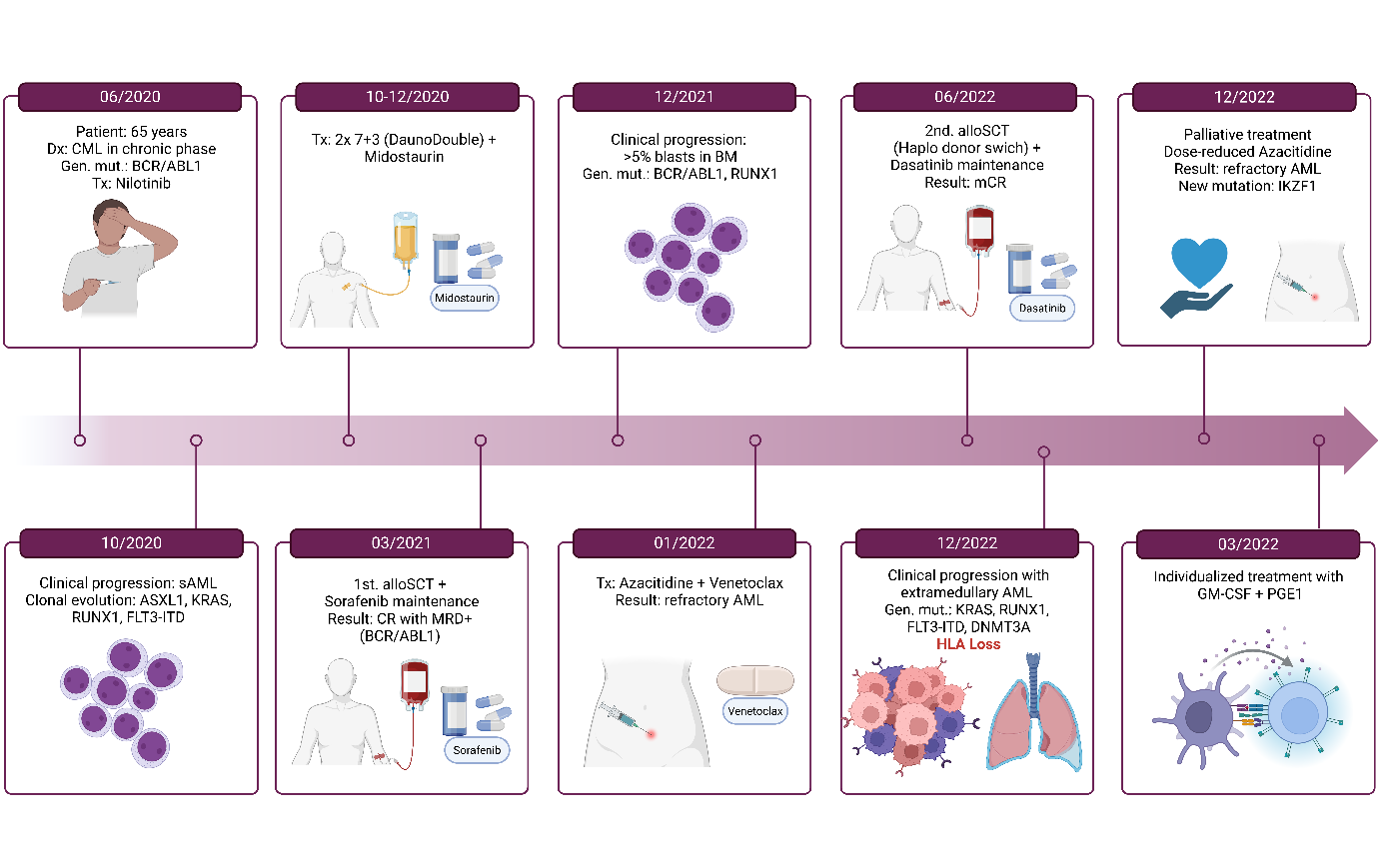
**

**Supplemental figure 1. Timeline of major clinical events until individualized treatment with GM-CSF + PEG1.**

*Dx:* diagnosis; *Gen. mut*: genetic mutation *Tx:* treatment; *CML:* chronic myeloid leukaemia; *sAML:* secondary acute myeloid leukaemia; *“2x 7+3”:* standard induction treatment with cytarabine 100mg/m2 days 1-7 and daunorubicin 60mg/m2 days 3-5, randomized in the “*double induction”* arm; *alloSCT:* allogeneic stem cell transplantation, *BM:* bone marrow; *CR:* complete remission; *MRD*: minimal residual disease, *mCR:* molecular CR; *GM-CSF:* Granulocyte-Macrophage Colony-Stimulating Factor; *PGE1:* Prostaglandin E1.

**Supplemental figure 2**


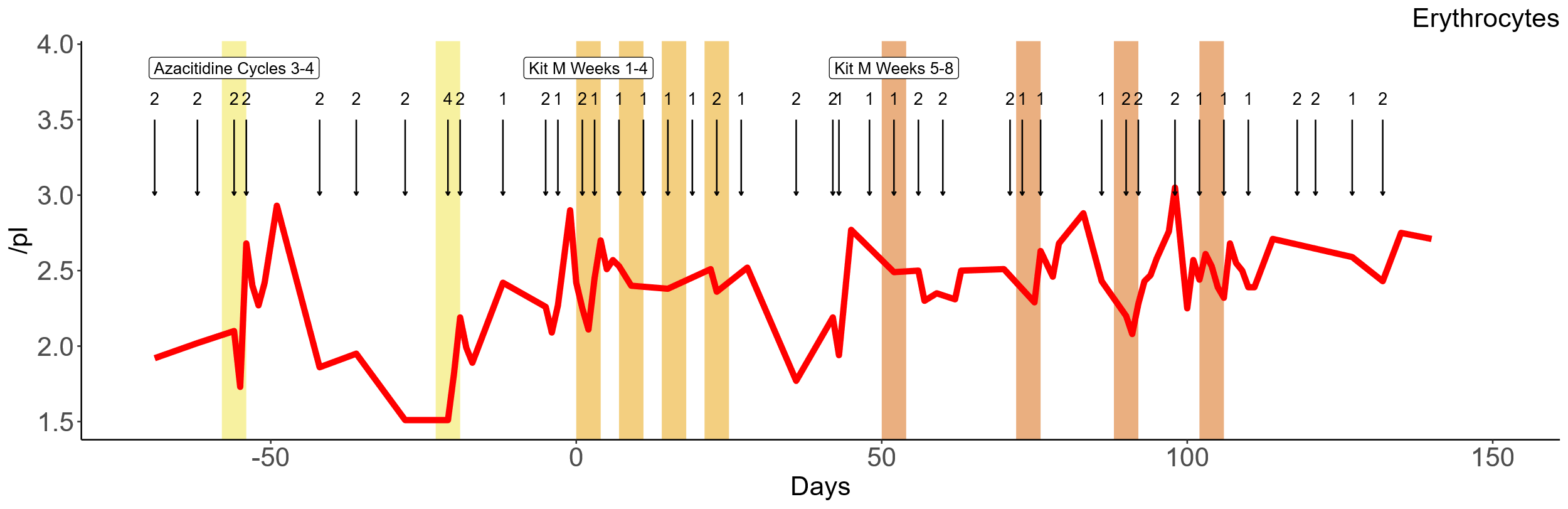


**Supplemental figure 2. Erythrocytes counts in peripheral blood and red blood cell transfusions during treatment course.**

The bars in colors represent treatment phases: every yellow bar indicate 1 cycle of Azacitidine; orange bars (starting on day 0) represent the ramp up phase of experimental treatment with Kit M (each bar indicate one five-day cycle with daily infusion of *Kit M*: GM-CSF+PEG1); brown bars (starting day 50) indicate the final dose phase of Kit M (each bar d1-5 with daily infusions). For more details on the treatment protocol see Table 1. The red line illustrates the course of Erythrocytes (/pl). Black arrows indicate blood transfusion, and the numbers above arrows the units transfused at that time point.

**Supplemental figure 3**


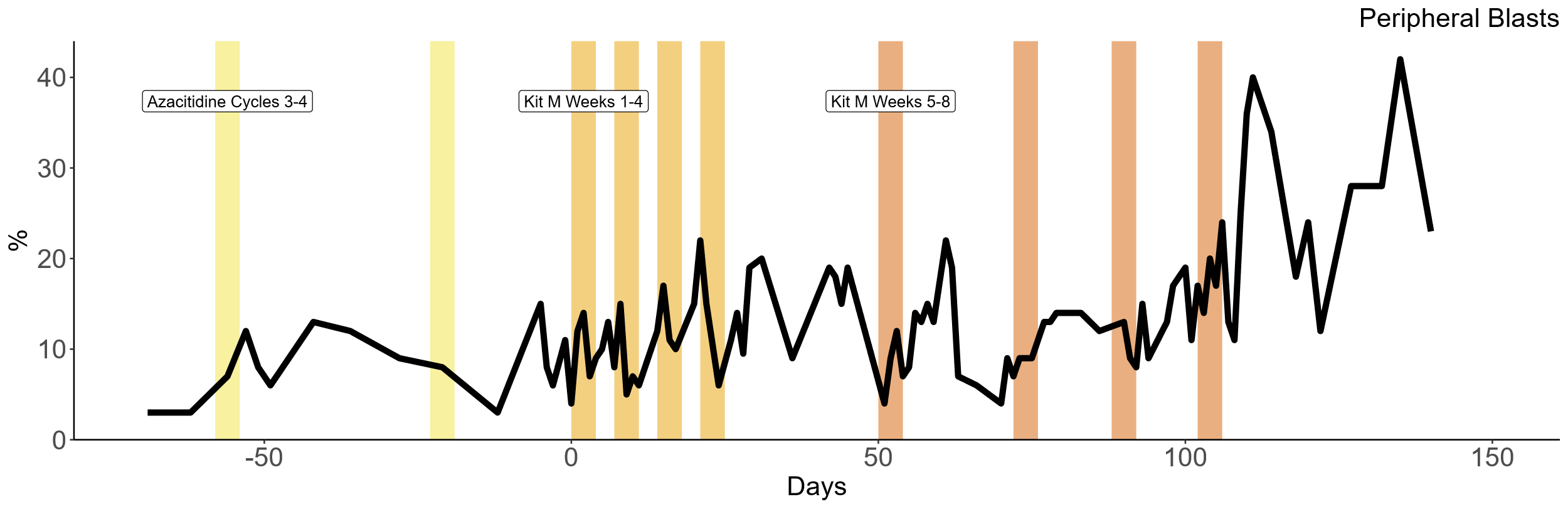
**Supplemental figure 3. Blast counts in peripheral blood during treatment course.**

The bars in colors represent treatment phases: every yellow bar indicate 1 cycle of Azacitidine; orange bars (starting on day 0) represent the ramp up phase of experimental treatment with Kit M (each bar indicate one five-day cycle with daily infusion of *Kit M*: GM-CSF+PEG1); brown bars (starting day 50) indicate the final dose phase of Kit M (each bar d1-5 with daily infusions). For more details on the treatment protocol see Supplemental Table 1. The black line illustrates the course of peripheral blasts (percentage of WBC).

**Supplemental figure 4**

**
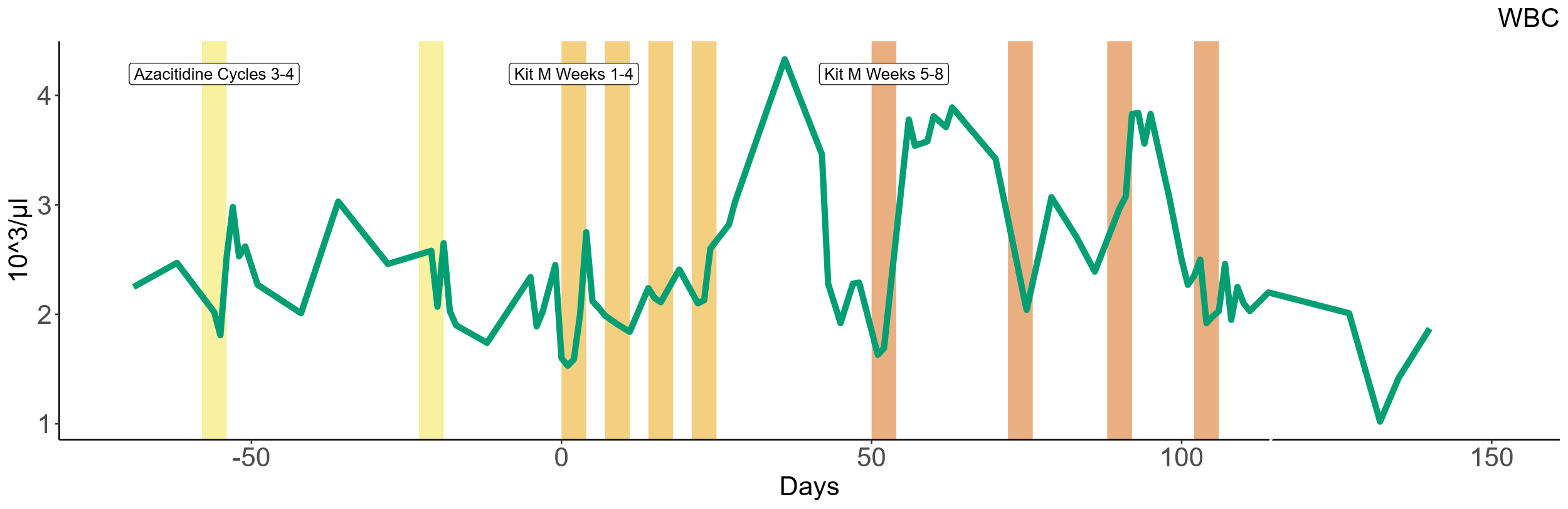
**

**Supplemental figure 4.** **White blood cell counts in peripheral during treatment course.**

The bars in colors represent treatment phases: every yellow bar indicate 1 cycle of Azacitidine; orange bars (starting on day 0) represent the ramp up phase of experimental treatment with Kit M (each bar indicate one five-day cycle with daily infusion of *Kit M*: GM-CSF+PEG1); brown bars (starting day 50) indicate the final dose phase of Kit M (each bar d1-5 with daily infusions). For more details on the treatment protocol see Table 1. The green line illustrates the course of white blood cells.

**Supplemental figure 5**

a. b.


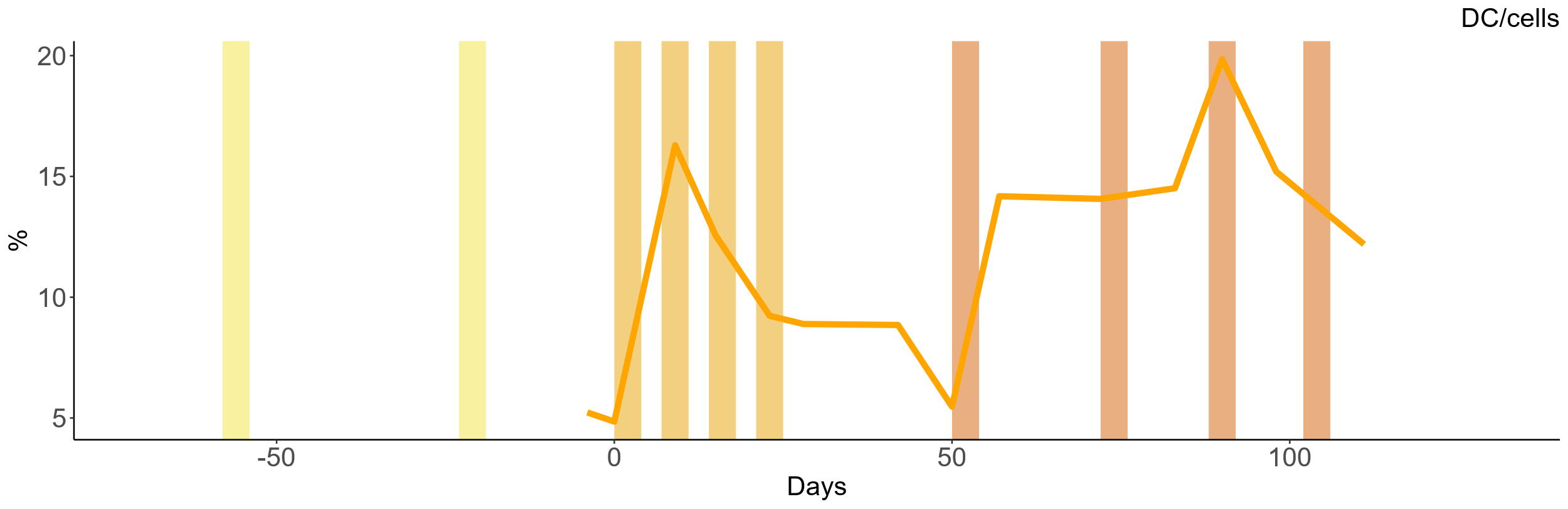

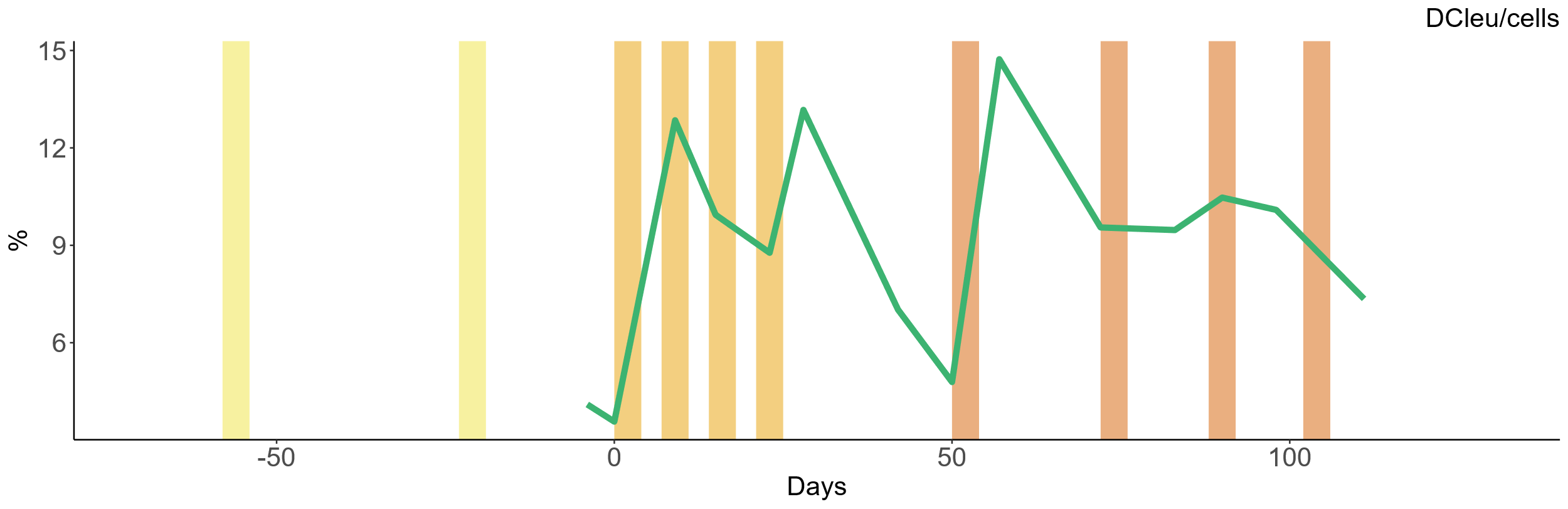


c.


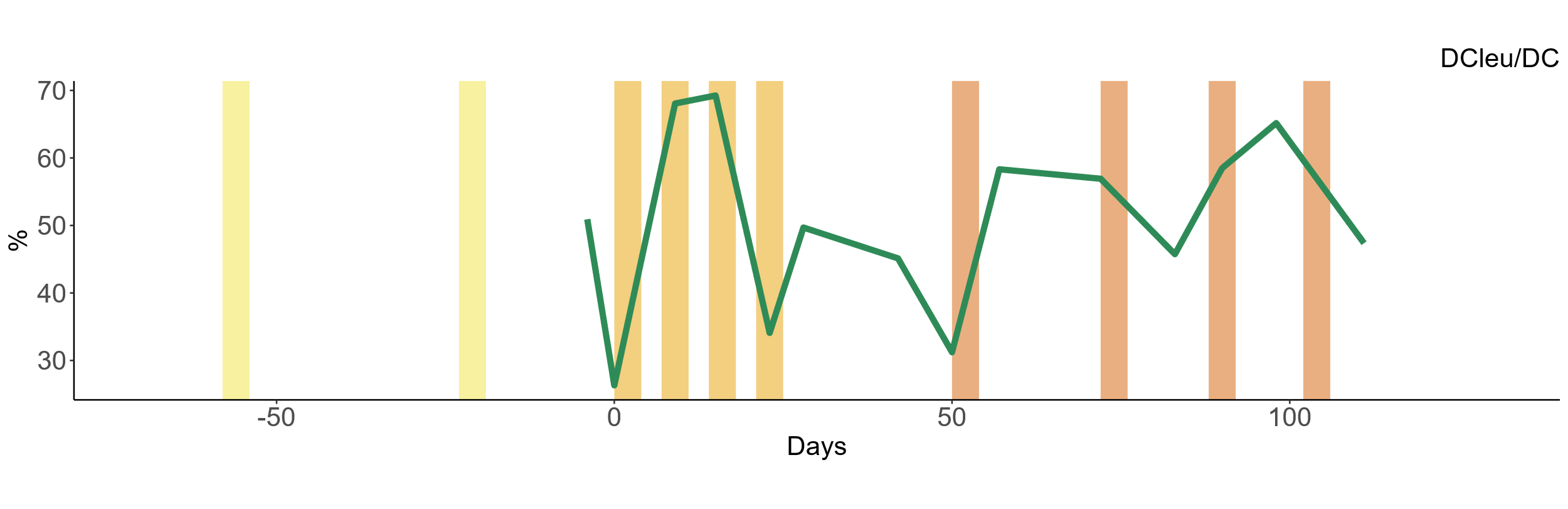


**Supplemental figure 5 a-c. Relative frequencies of dendritic cells (DC) and dendritic cells of leukaemic origin in peripheral blood during treatment course.**

a. Dendritic Cells (DC), b. Dendritic Cells of leukaemic origin (DCleu) relative to all cells, and c. DCleu relative to all DCs.

The colored lines illustrate the course of relative frequencies (%) of the given cell subtype measured at different time points during treatment course. For more details on the treatment phases please see legend to supplemental figure 1-3.

**Supplemental figure 6**

a. b.


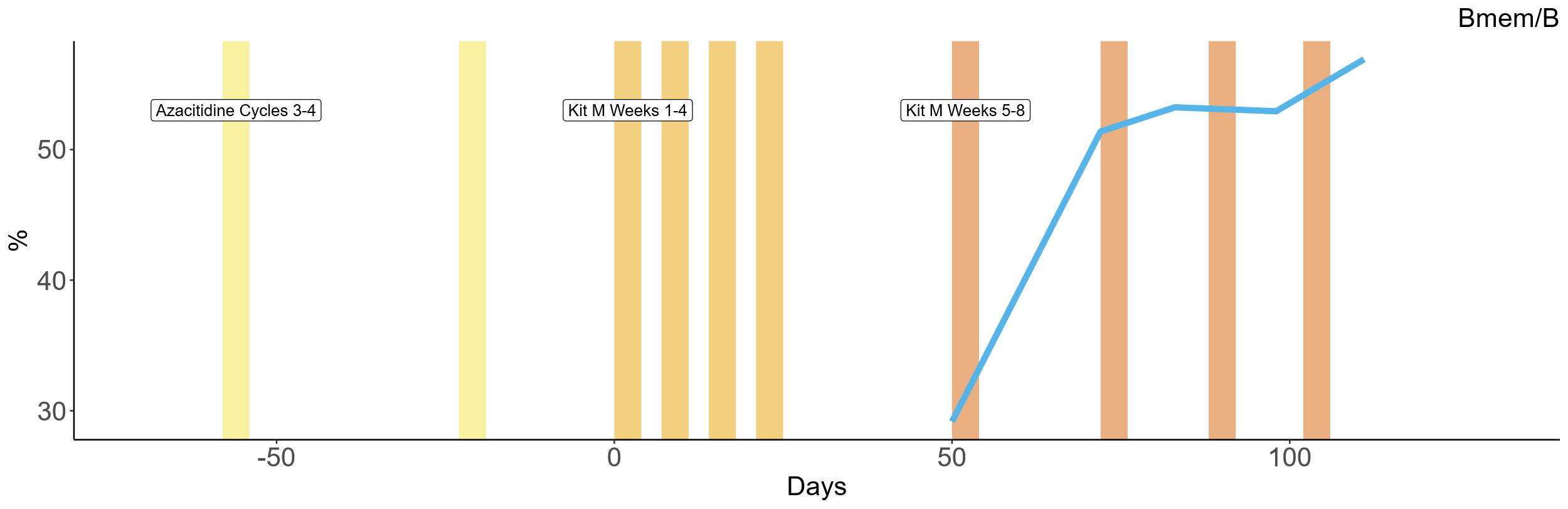

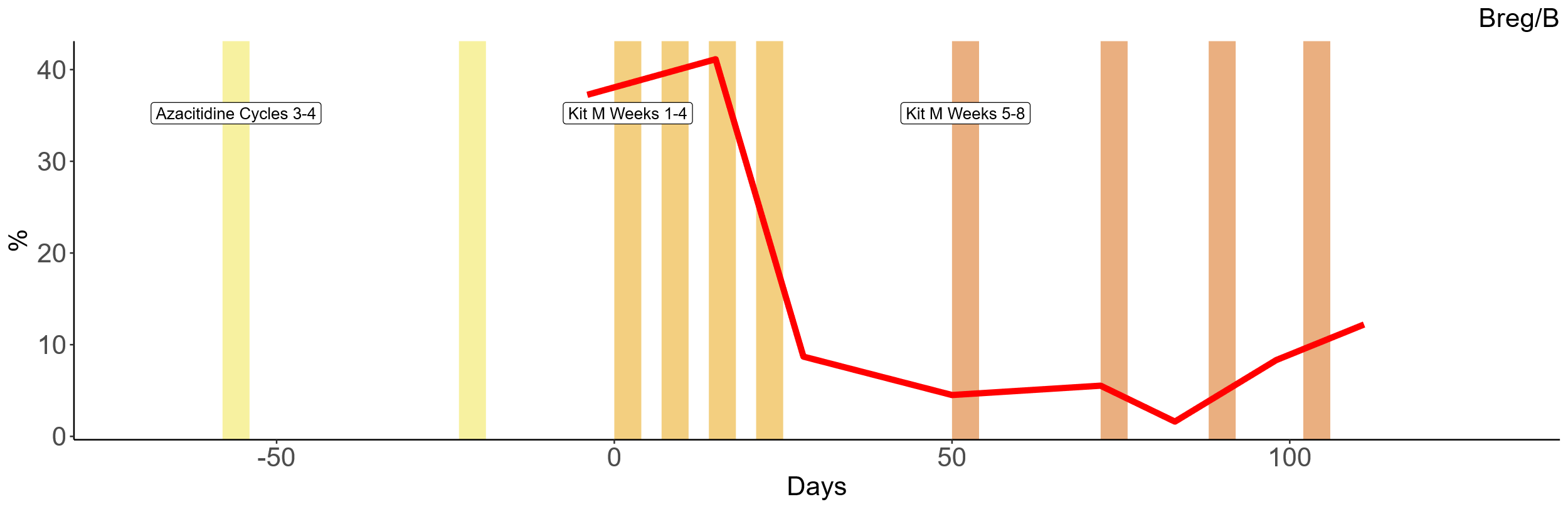


**Supplemental figure 6 a-b. Relative frequencies of B cells in peripheral blood during treatment course.**

a. memory B cells (Bmem), b. regulatory B cells (Breg)

The colored lines illustrate the course of relative frequencies (%) of the given cell subtype measured at different time points during treatment course. For more details on the treatment phases please see legend to supplemental figure 1-3.

| Cell Type  **Supplemental Table 1:** Cell subtypes | | Name of Subgroups | Abbreviation of Subgroups | Surface Marker |
| --- | --- | --- | --- | --- |
| Blast cells | | Leukemic blasts | Bla^*^ | Bla^+^ (e.g. CD34^+^, CD177^+^) |
|  | | Proliferating blasts | Bla_prol_ | Bla^+^DC^-^CD71^+^ |
|  | | Checkpointmarker (PD1^+^, 2B4^+^TIM3^+^,CTLA4^+^) expressing Blasts | Bla_PD1_^*^  Bla_2B4_^*^  Bla_TIM3_^*^  Bla_CTLA4_^*^ | Bla^+^CD279^+^  Bla^+^2B4^+^  Bla^+^TIM3^+^  Bla^+^CD152^+^ |
| Dendritic cells   \|  \| \| --- \| \|  \| \|  \| \|  \| | | Dendritic cells | DC^*^ | DC^+^ (CD80^+^, CD206^+^) |
|  |  | Leukemia derived DC | DC_leu_^*^ | DC^+^Bla^+^ |
|  |  | Mature DC | DC_mat_^*^ | DC^+^CD197^+^ |
| T cells | | CD3^+^ pan T cells | T3 | CD3^+^ |
|  |  | Naive T cells | T3n | CD3^+^CD45RO^-^ |
|  |  | Non-naive T cells | T3nn | CD3^+^CD45RO^+^ |
|  |  | Central (memory) T cells | T3cm | CD3^+^CD45RO^+^CD197^+^ |
|  |  | Effector (memory) T cells | T3em | CD3^+^CD45RO^-^CD197^+^ |
|  |  | Integrinβ7^+^ coexpressing T cells | T3Intβ7* | CD3^+^Integrinβ7^+^ |
|  |  | Regulatory T cells | Treg* | CD3^+^CD4^+^CD25^+^CD127^low^ |
|  | | T-helper 1 | TH1^*^ | CD4^+^CXCR3^+^ |
|  | | T-helper 2 | TH2^*^ | CD4^+^CCR4^+^CCR6^-^ |
|  | | T-helper 17 | TH17^*^ | CD4^+^CCR4^+^CCR6^+^ |
|  | | Checkpointmarker expressing Tcells | T3_PD1_^*^  T3_2B4_^*^  T3_CTLA4_^*^  T3_TIGIT_^*^  T3_KLRG_^*^  T3_TIM3_^*^ | CD3^+^CD279^+^  CD3^+^2B4^+^  CD3^+^CD152^+^  CD3^+^TIGIT^+^  CD3^+^KLRG^+^  CD3^+^TIM3^+^ |
| B cells | | CD19^+^ pan B cells | B | CD19^+^ |
|  | | Regulatory B cells | Breg* | CD19^+^CD24^++^CD38^++^ |
|  | | B-memory cells | Bmem* | CD19^+^CD24^++^CD27^+^ |
|  | | Immigrated B- memory cells | Bimm.mem* | CD19^+^IgD^-^CD27^+^CD183^+^ |
| Natural killer cells | | CD3^-^CD56^+^NK cells | NK^*^ | CD3^-^CD56^+^ |
| Invariant natural killer T cells | | 6B11^+^ iNKT cells | iNKT^*^ | 6B11^+^ |
|  |  |  |  |  |
| T cells | | CD3^+^ pan T cells | \| γT3^*^ \| \| --- \| | IFNγ^+^CD3^+^ |
|  |  | Non-naive T cells | γT3nn^*^ | IFNγ^+^CD3^+^CD45RO+ |
|  |  | Central (memory) T cells | γT3cm^*^ | IFNγ^+^CD3^+^CD45RO^+^CD197^+^ |
|  |  | Integrinβ7^+^ coexpressing T cells | γT3Intβ7 | IFNγ^+^CD3^+^β7^+^ |
|  | | Regulatory T cells | γTreg* | IFNγ^+^CD4^+^CD25^+^CD127^low^ |
| NK cells | | CD3^-^CD56^+^NK cells | γNK | IFNγ^+^CD3^-^CD56^+^ |
| T cells | | CD3^+^ pan T cells | degT3^*^ | CD107a^+^CD3^+^ |
|  |  | Non-naive T cells | degT3nn | CD107a^+^CD3^+^CD45RO+ |
|  |  | Central (memory) T cells | degT3cm | CD107a^+^CD3^+^CD45RO^+^CD197^+^ |
|  |  | Integrinβ7^+^ coexpressing T cells | degT3Intβ7 | CD107a^+^CD3^+^Integrinβ7^+^ |
|  |  | Regulatory T cells | degTreg | CD107a^+^CD4^+^CD25^+^CD127^low^ |
| B cells | | CD19^+^ pan B cells | degB | CD107a^+^CD19^+^ |
|  | | Regulatory B cells | degB_reg_ | CD107a^+^CD19^+^CD24^++^CD38^++^ |
| NK cells | | CD3^-^CD56^+^NK cells | degNK^*^ | CD107a^+^CD3^-^CD56^+^ |

*subtypes reported in the letter
